# 3-[(2-Chloro-6-methyl­quinolin-3-yl)meth­yl]quinazolin-4(3*H*)-one

**DOI:** 10.1107/S1600536810020830

**Published:** 2010-06-05

**Authors:** S. Mohana Roopan, F. Nawaz Khan, Sriramakrishnaswamy Kone, Venkatesha R. Hathwar, Mehmet Akkurt

**Affiliations:** a Organic and Medicinal Chemistry Research Laboratory, Organic Chemistry Division, School of Advanced Sciences, VIT University, Vellore 632 014, Tamil Nadu, India; b Solid State and Structural Chemistry Unit, Indian Institute of Science, Bangalore 560 012, Karnataka, India; c Department of Physics, Faculty of Arts and Sciences, Erciyes University, 38039 Kayseri, Turkey

## Abstract

In the title mol­ecule, C_19_H_14_ClN_3_O, the quinoline and quinazoline ring systems form a dihedral angle of 80.75 (4)°. In the crystal, the mol­ecules are linked by pairs of C—H⋯N hydrogen bonds into centrosymmetric dimers, generating *R*_2_^2^(6) ring motifs. The structure is further stabilized by C—H⋯π inter­actions and π–π stacking inter­actions [centroid–centroid distances = 3.7869 (8) and 3.8490 (8) Å].

## Related literature

For quinoline analogues, see: Roopan *et al.* (2009 ▶); Khan *et al.* (2009 ▶, 2010*a* ▶,*b* ▶). For quinazolinone analogues, see: Roopan *et al.* (2008*a* ▶,*b* ▶). For the properties and applications of related compounds, see: Abdel-Hamide *et al.* (1996 ▶); Bekhit & Khalil (1998 ▶); Chapman *et al.* (1963 ▶); Honda *et al.* (1979 ▶).
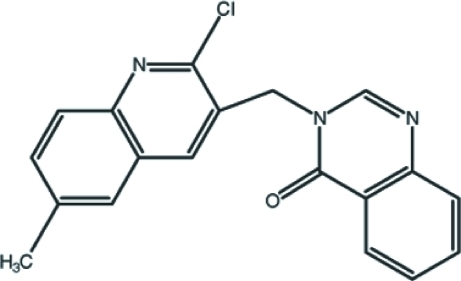


## Experimental

### 

#### Crystal data

C_19_H_14_ClN_3_O
*M* *_r_* = 335.78
Monoclinic, 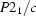

*a* = 7.86728 (14) Å
*b* = 14.7098 (3) Å
*c* = 13.7055 (3) Å
β = 102.1500 (17)°
*V* = 1550.56 (5) Å^3^
*Z* = 4
Mo *K*α radiation
μ = 0.26 mm^−1^
*T* = 295 K
0.25 × 0.21 × 0.16 mm

#### Data collection

Oxford Diffraction Xcalibur E CCD diffractometer
Absorption correction: multi-scan (*CrysAlis PRO*; Oxford Diffraction, 2009 ▶) *T*_min_ = 0.938, *T*_max_ = 0.960
15755 measured reflections
3048 independent reflections
2417 reflections with *I* > 2σ(*I*)
*R*_int_ = 0.026

#### Refinement

*R*[*F* ^2^ > 2σ(*F* ^2^)] = 0.034
*wR*(*F* ^2^) = 0.096
*S* = 1.14
3048 reflections
219 parameters
H-atom parameters constrained
Δρ_max_ = 0.18 e Å^−3^
Δρ_min_ = −0.19 e Å^−3^

### 

Data collection: *CrysAlis PRO* (Oxford Diffraction, 2009 ▶); cell refinement: *CrysAlis PRO*; data reduction: *CrysAlis PRO*; program(s) used to solve structure: *SHELXS97* (Sheldrick, 2008 ▶); program(s) used to refine structure: *SHELXL97* (Sheldrick, 2008 ▶); molecular graphics: *ORTEP-3* (Farrugia, 1997 ▶); software used to prepare material for publication: *WinGX* (Farrugia, 1999 ▶).

## Supplementary Material

Crystal structure: contains datablocks global, I. DOI: 10.1107/S1600536810020830/gk2276sup1.cif

Structure factors: contains datablocks I. DOI: 10.1107/S1600536810020830/gk2276Isup2.hkl

Additional supplementary materials:  crystallographic information; 3D view; checkCIF report

## Figures and Tables

**Table 1:** Hydrogen-bond geometry (Å, °) *Cg*1 and *Cg*2 are the centroids of the N1/C1–C4/C9 and N2/N3/C12/C13/C18/C19 rings, respectively.

*D*—H⋯*A*	*D*—H	H⋯*A*	*D*⋯*A*	*D*—H⋯*A*
C19—H19⋯N3^i^	0.93	2.51	3.271 (2)	139
C8—H8⋯*Cg*2^ii^	0.93	2.89	3.6598 (16)	142
C10—H10*A*⋯*Cg*1^iii^	0.96	2.68	3.5189 (17)	146

Symmetry codes: (i) 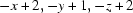; (ii) 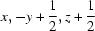; (iii) 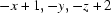.

**Fig. 1.**
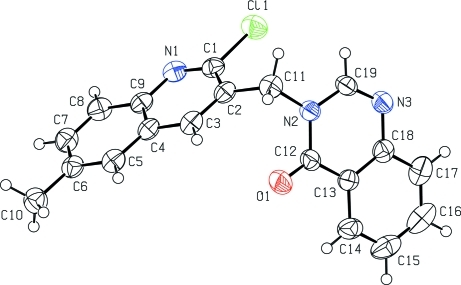
The molecular structure of the title compound, showing the atom-numbering scheme and displacement ellipsoids drawn at the 50% probability level.

**Fig. 2.**
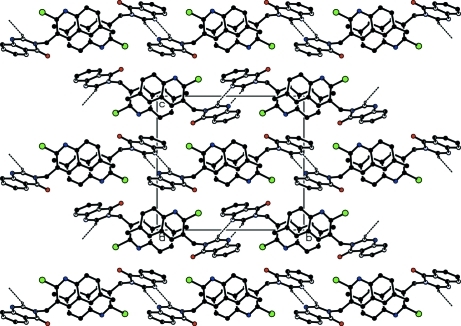
Crystal packing viewed down a axis. H atoms not involved in the intermolecular interactions (dashed lines) have been omitted for clarity.

## supplementary crystallographic information

### Comment

Heterocyclic chemistry comprises at least half of all organic chemistry research worldwide (Roopan *et al.*, 2008*a*,b). In particular, heterocyclic structures form the basis of many pharmaceutical, agrochemical and veterinary products. 4(3*H*)-quinazolinones and quinolines (Roopan *et al.*, 2009) are classes of fused heterocycles that are of considerable interest because of their biological properties. Some are endowed with antimicrobial, aniconvulsant, antihistamine and anti-inflammatory properties (Abdel-Hamide *et al.*, 1996, Chapman *et al.*, 1963, Bekhit *et al.*, 1998). On the other hand, some quinoline derivatives also have various biological properties like antioxidant, hemolytic and cytotoxicity. These observations prompted us to synthesized heterocyclic compounds containing a quinolinyl-quinazolinone moiety.

As shown in Fig. 1, the quinoline (N1/C1–C9) and quinazoline (N2/N3/C12–C19) ring systems of the title molecule (I) are almost planar with maximum deviations of -0.016 (1) Å for C2 and 0.065 (1) Å for N2, respectively, and there is a dihedral angle of 80.75 (4)° between them.

Two neighbouring molecules are linked by a pair of C—H···N hydrogen bonds into a pseudo-centrosymmetric dimer, generating an *R*^2^_2_(6) ring motif (Table 1, Fig. 2). In addition, the structure is stabilized by C—H···π interactions (Table 1) and π-π stacking interactions [*Cg*1···*Cg*3(2 - *x*,-*y*, 2 - *z*) = 3.7869 (8) Å and *Cg*3···*Cg*3(1 - *x*, -*y*, 2 - *z*) = 3.8490 (8) Å; where *Cg*1 and *Cg*3 are centroids of the N1/C1–C4/C9 and C4–C9 rings, respectively].

### Experimental

To a solution of 4(3*H*)-quinazolinone (146 mg, 1 mmol) in 2 ml of DMF were added KOtBu (112 mg, 1 mmol) in 10 ml of THF and 2-chloro-3-(chloromethyl)-6-methylquinoline (225 mg, 1 mmol) and the resulting mixture was refluxed at 343 K for 1 h. After the completion, the reaction was cooled and the excess of solvent removed under reduced pressure. Crushed ice was mixed with the residue. White solid was formed which was purified by column chromatography using hexane and ethylacetate as the eluant. Crystals of suitable quality were grown by solvent evaporation from a solution of the compound in diethyl ether.

### Refinement

The H atoms were positioned geometrically with C—H = 0.93, 0.97 and 0.96 Å, for aromatic, methylene and methyl H, respectively, and constrained to ride on their parent atoms, with *U*_iso_(H) = *x*U_eq_(C), where *x* = 1.5 for methyl H, and *x* = 1.2 for others H atoms.

### Crystal data

**Table:**

C_19_H_14_ClN_3_O	*F*(000) = 696
*M**_r_* = 335.78	*D*_x_ = 1.438 Mg m^−^^3^
Monoclinic, *P*2_1_/*c*	Mo *K*α radiation, λ = 0.71073 Å
Hall symbol: -P 2ybc	Cell parameters from 1326 reflections
*a* = 7.86728 (14) Å	θ = 2.0–20.7°
*b* = 14.7098 (3) Å	µ = 0.26 mm^−^^1^
*c* = 13.7055 (3) Å	*T* = 295 K
β = 102.1500 (17)°	Needle, colourless
*V* = 1550.56 (5) Å^3^	0.25 × 0.21 × 0.16 mm
*Z* = 4	

### Data collection

**Table:**

Oxford Diffraction Xcalibur E CCD diffractometer	3048 independent reflections
Radiation source: Enhance (Mo) X-ray Source	2417 reflections with *I* > 2σ(*I*)
graphite	*R*_int_ = 0.026
ω scans	θ_max_ = 26.0°, θ_min_ = 2.7°
Absorption correction: multi-scan (*CrysAlis PRO*; Oxford Diffraction, 2009)	*h* = −9→9
*T*_min_ = 0.938, *T*_max_ = 0.960	*k* = −18→18
15755 measured reflections	*l* = −16→16

### Refinement

**Table:**

Refinement on *F*^2^	Secondary atom site location: difference Fourier map
Least-squares matrix: full	Hydrogen site location: inferred from neighbouring sites
*R*[*F*^2^ > 2σ(*F*^2^)] = 0.034	H-atom parameters constrained
*wR*(*F*^2^) = 0.096	*w* = 1/[σ^2^(*F*_o_^2^) + (0.0511*P*)^2^ + 0.0739*P*] where *P* = (*F*_o_^2^ + 2*F*_c_^2^)/3
*S* = 1.14	(Δ/σ)_max_ = 0.001
3048 reflections	Δρ_max_ = 0.18 e Å^−^^3^
219 parameters	Δρ_min_ = −0.19 e Å^−^^3^
0 restraints	Extinction correction: *SHELXL97* (Sheldrick, 2008), FC^*^=KFC[1+0.001XFC^2^Λ^3^/SIN(2Θ)]^-1/4^
Primary atom site location: structure-invariant direct methods	Extinction coefficient: 0.0123 (15)

### Special details

**Table:**

Geometry. Bond distances, angles *etc*. have been calculated using the rounded fractional coordinates. All su's are estimated from the variances of the (full) variance-covariance matrix. The cell e.s.d.'s are taken into account in the estimation of distances, angles and torsion angles
Refinement. Refinement on *F*^2^ for ALL reflections except those flagged by the user for potential systematic errors. Weighted *R*-factors *wR* and all goodnesses of fit *S* are based on *F*^2^, conventional *R*-factors *R* are based on *F*, with *F* set to zero for negative *F*^2^. The observed criterion of *F*^2^ > σ(*F*^2^) is used only for calculating -*R*-factor-obs *etc*. and is not relevant to the choice of reflections for refinement. *R*-factors based on *F*^2^ are statistically about twice as large as those based on *F*, and *R*-factors based on ALL data will be even larger.

### Fractional atomic coordinates and isotropic or equivalent isotropic displacement parameters (Å^2^)

**Table:**

	*x*	*y*	*z*	*U*_iso_*/*U*_eq_	
Cl1	1.05099 (5)	0.28142 (3)	1.12633 (3)	0.0552 (2)	
O1	0.60460 (15)	0.24457 (8)	0.79970 (8)	0.0550 (4)	
N1	0.90795 (14)	0.12379 (9)	1.13506 (8)	0.0409 (4)	
N2	0.82439 (14)	0.32871 (8)	0.89095 (8)	0.0368 (4)	
N3	0.79403 (17)	0.48498 (9)	0.92418 (9)	0.0453 (4)	
C1	0.94294 (18)	0.18353 (10)	1.07237 (11)	0.0389 (5)	
C2	0.90272 (18)	0.17726 (10)	0.96669 (10)	0.0374 (5)	
C3	0.82067 (18)	0.09933 (10)	0.92894 (10)	0.0395 (5)	
C4	0.77799 (17)	0.03094 (10)	0.99195 (10)	0.0366 (4)	
C5	0.69103 (18)	−0.05001 (10)	0.95563 (11)	0.0413 (5)	
C6	0.64859 (18)	−0.11435 (10)	1.01883 (11)	0.0403 (5)	
C7	0.6946 (2)	−0.09747 (11)	1.12254 (11)	0.0461 (5)	
C8	0.77957 (19)	−0.02054 (11)	1.16013 (11)	0.0444 (5)	
C9	0.82367 (17)	0.04583 (10)	1.09569 (10)	0.0373 (5)	
C10	0.5554 (2)	−0.20015 (11)	0.97943 (13)	0.0519 (6)	
C11	0.9436 (2)	0.25093 (11)	0.89857 (12)	0.0442 (5)	
C12	0.65169 (19)	0.31653 (10)	0.84072 (10)	0.0383 (4)	
C13	0.54199 (18)	0.39518 (10)	0.84513 (10)	0.0376 (5)	
C14	0.3617 (2)	0.38997 (12)	0.80976 (11)	0.0497 (6)	
C15	0.2604 (2)	0.46424 (15)	0.81376 (12)	0.0613 (7)	
C16	0.3351 (3)	0.54611 (15)	0.85043 (13)	0.0658 (7)	
C17	0.5113 (2)	0.55297 (12)	0.88491 (12)	0.0571 (6)	
C18	0.61654 (19)	0.47690 (10)	0.88456 (10)	0.0406 (5)	
C19	0.88460 (19)	0.41208 (11)	0.92637 (11)	0.0415 (5)	
H3	0.79220	0.09100	0.86020	0.0470*	
H5	0.66180	−0.06000	0.88710	0.0500*	
H7	0.66590	−0.14020	1.16640	0.0550*	
H8	0.80880	−0.01170	1.22880	0.0530*	
H10A	0.44010	−0.19950	0.99230	0.0780*	
H10B	0.61780	−0.25170	1.01190	0.0780*	
H10C	0.54880	−0.20420	0.90880	0.0780*	
H11A	1.06170	0.27200	0.92330	0.0530*	
H11B	0.93730	0.22560	0.83250	0.0530*	
H14	0.31130	0.33570	0.78350	0.0600*	
H15	0.14040	0.46010	0.79180	0.0740*	
H16	0.26500	0.59680	0.85160	0.0790*	
H17	0.56050	0.60830	0.90850	0.0690*	
H19	1.00250	0.41650	0.95480	0.0500*	

### Atomic displacement parameters (Å^2^)

**Table:**

	*U*^11^	*U*^22^	*U*^33^	*U*^12^	*U*^13^	*U*^23^
Cl1	0.0554 (3)	0.0545 (3)	0.0522 (3)	−0.0078 (2)	0.0032 (2)	−0.0056 (2)
O1	0.0623 (7)	0.0417 (7)	0.0540 (7)	−0.0106 (5)	−0.0034 (5)	−0.0054 (5)
N1	0.0375 (7)	0.0482 (8)	0.0363 (7)	0.0036 (6)	0.0060 (5)	−0.0013 (6)
N2	0.0353 (6)	0.0375 (7)	0.0368 (6)	−0.0034 (5)	0.0059 (5)	0.0015 (5)
N3	0.0483 (8)	0.0392 (8)	0.0451 (7)	−0.0082 (6)	0.0021 (6)	0.0015 (6)
C1	0.0316 (7)	0.0426 (9)	0.0422 (8)	0.0051 (6)	0.0068 (6)	−0.0016 (7)
C2	0.0347 (8)	0.0412 (9)	0.0377 (8)	0.0094 (6)	0.0110 (6)	0.0016 (6)
C3	0.0442 (8)	0.0435 (9)	0.0320 (7)	0.0103 (7)	0.0109 (6)	−0.0005 (6)
C4	0.0365 (8)	0.0397 (8)	0.0347 (7)	0.0109 (6)	0.0100 (6)	0.0001 (6)
C5	0.0440 (8)	0.0446 (9)	0.0361 (8)	0.0083 (7)	0.0105 (6)	−0.0042 (7)
C6	0.0358 (8)	0.0401 (9)	0.0468 (9)	0.0088 (6)	0.0128 (6)	0.0002 (7)
C7	0.0449 (9)	0.0495 (10)	0.0467 (9)	0.0038 (7)	0.0158 (7)	0.0083 (7)
C8	0.0439 (8)	0.0557 (10)	0.0341 (8)	0.0024 (7)	0.0096 (6)	0.0028 (7)
C9	0.0326 (7)	0.0436 (9)	0.0369 (8)	0.0087 (6)	0.0099 (6)	0.0007 (6)
C10	0.0519 (10)	0.0455 (10)	0.0604 (10)	0.0026 (8)	0.0169 (8)	−0.0015 (8)
C11	0.0436 (9)	0.0479 (9)	0.0436 (8)	0.0035 (7)	0.0152 (7)	0.0017 (7)
C12	0.0430 (8)	0.0383 (8)	0.0317 (7)	−0.0078 (7)	0.0038 (6)	0.0041 (6)
C13	0.0392 (8)	0.0436 (9)	0.0293 (7)	−0.0041 (7)	0.0054 (6)	0.0083 (6)
C14	0.0414 (9)	0.0646 (11)	0.0400 (9)	−0.0059 (8)	0.0015 (7)	0.0123 (8)
C15	0.0433 (10)	0.0941 (15)	0.0460 (10)	0.0117 (10)	0.0084 (7)	0.0210 (10)
C16	0.0701 (13)	0.0799 (14)	0.0482 (10)	0.0341 (11)	0.0143 (9)	0.0114 (10)
C17	0.0739 (12)	0.0484 (10)	0.0478 (10)	0.0125 (9)	0.0101 (8)	0.0022 (8)
C18	0.0475 (9)	0.0425 (9)	0.0318 (7)	−0.0007 (7)	0.0083 (6)	0.0046 (6)
C19	0.0386 (8)	0.0432 (9)	0.0402 (8)	−0.0114 (7)	0.0028 (6)	0.0039 (7)

### Geometric parameters (Å, °)

**Table:**

Cl1—C1	1.7545 (15)	C13—C14	1.401 (2)
O1—C12	1.2191 (19)	C13—C18	1.396 (2)
N1—C1	1.2984 (19)	C14—C15	1.360 (3)
N1—C9	1.3767 (19)	C15—C16	1.387 (3)
N2—C11	1.469 (2)	C16—C17	1.371 (3)
N2—C12	1.3994 (19)	C17—C18	1.393 (2)
N2—C19	1.367 (2)	C3—H3	0.9300
N3—C18	1.393 (2)	C5—H5	0.9300
N3—C19	1.284 (2)	C7—H7	0.9300
C1—C2	1.419 (2)	C8—H8	0.9300
C2—C3	1.363 (2)	C10—H10A	0.9600
C2—C11	1.509 (2)	C10—H10B	0.9600
C3—C4	1.412 (2)	C10—H10C	0.9600
C4—C5	1.411 (2)	C11—H11A	0.9700
C4—C9	1.4084 (19)	C11—H11B	0.9700
C5—C6	1.371 (2)	C14—H14	0.9300
C6—C7	1.413 (2)	C15—H15	0.9300
C6—C10	1.502 (2)	C16—H16	0.9300
C7—C8	1.359 (2)	C17—H17	0.9300
C8—C9	1.408 (2)	C19—H19	0.9300
C12—C13	1.452 (2)		
			
Cl1···N2	3.4130 (12)	C15···H3^ix^	2.9900
Cl1···C19	3.3776 (16)	C16···H3^ix^	2.9200
Cl1···H11A	2.8000	C18···H8^vi^	2.9100
Cl1···H19	3.0400	C19···H15^x^	3.0800
Cl1···H16^i^	3.1300	C19···H19^iv^	3.0300
Cl1···H11B^ii^	3.1400	C19···H8^vi^	3.0300
O1···C2	3.0757 (18)	H3···O1	2.7300
O1···C3	3.0535 (18)	H3···H5	2.5100
O1···H3	2.7300	H3···H11B	2.3600
O1···H11B	2.5800	H3···C15^xi^	2.9900
O1···H14	2.6400	H3···C16^xi^	2.9200
O1···H7^iii^	2.7400	H5···H3	2.5100
N2···Cl1	3.4130 (12)	H5···H10C	2.3400
N3···C19^iv^	3.271 (2)	H5···C14^xi^	2.7700
N1···H15^v^	2.8000	H5···C15^xi^	2.9600
N3···H19^iv^	2.5100	H7···O1^iii^	2.7400
N3···H8^vi^	2.7300	H8···N3^ii^	2.7300
C1···C5^vii^	3.573 (2)	H8···C18^ii^	2.9100
C2···O1	3.0757 (18)	H8···C19^ii^	3.0300
C3···O1	3.0535 (18)	H10A···C1^iii^	2.9700
C3···C9^vii^	3.592 (2)	H10A···C2^iii^	2.8900
C3···C12	3.572 (2)	H10A···C3^iii^	2.9100
C4···C4^vii^	3.5715 (19)	H10A···C4^iii^	3.0500
C4···C6^iii^	3.547 (2)	H10A···C12^iii^	3.0700
C5···C1^vii^	3.572 (2)	H10B···H17^viii^	2.4900
C6···C4^iii^	3.547 (2)	H10B···H11A^vii^	2.5100
C9···C3^vii^	3.592 (2)	H10C···H5	2.3400
C12···C3	3.572 (2)	H11A···Cl1	2.8000
C16···C18^i^	3.587 (2)	H11A···H19	2.2400
C17···C18^i^	3.539 (2)	H11A···H10B^vii^	2.5100
C17···C17^i^	3.557 (2)	H11B···O1	2.5800
C18···C17^i^	3.539 (2)	H11B···H3	2.3600
C18···C16^i^	3.587 (2)	H11B···Cl1^vi^	3.1400
C19···N3^iv^	3.271 (2)	H14···O1	2.6400
C19···Cl1	3.3776 (16)	H15···C19^xii^	3.0800
C19···C19^iv^	3.538 (2)	H15···N1^xiii^	2.8000
C1···H10A^iii^	2.9700	H16···Cl1^i^	3.1300
C2···H10A^iii^	2.8900	H17···C10^xiv^	2.9800
C3···H10A^iii^	2.9100	H17···H10B^xiv^	2.4900
C4···H10A^iii^	3.0500	H19···Cl1	3.0400
C10···H17^viii^	2.9800	H19···H11A	2.2400
C12···H10A^iii^	3.0700	H19···N3^iv^	2.5100
C14···H5^ix^	2.7700	H19···C19^iv^	3.0300
C15···H5^ix^	2.9600		
			
C1—N1—C9	117.14 (12)	C16—C17—C18	119.90 (17)
C11—N2—C12	118.32 (12)	N3—C18—C13	121.98 (13)
C11—N2—C19	120.35 (12)	N3—C18—C17	118.56 (14)
C12—N2—C19	121.18 (12)	C13—C18—C17	119.46 (14)
C18—N3—C19	116.34 (13)	N2—C19—N3	126.29 (14)
Cl1—C1—N1	115.34 (11)	C2—C3—H3	119.00
Cl1—C1—C2	117.89 (11)	C4—C3—H3	119.00
N1—C1—C2	126.78 (14)	C4—C5—H5	119.00
C1—C2—C3	115.35 (13)	C6—C5—H5	119.00
C1—C2—C11	123.66 (13)	C6—C7—H7	119.00
C3—C2—C11	120.99 (13)	C8—C7—H7	119.00
C2—C3—C4	121.45 (13)	C7—C8—H8	120.00
C3—C4—C5	123.08 (13)	C9—C8—H8	120.00
C3—C4—C9	117.63 (13)	C6—C10—H10A	110.00
C5—C4—C9	119.29 (13)	C6—C10—H10B	109.00
C4—C5—C6	121.62 (13)	C6—C10—H10C	109.00
C5—C6—C7	118.00 (14)	H10A—C10—H10B	109.00
C5—C6—C10	121.23 (14)	H10A—C10—H10C	109.00
C7—C6—C10	120.78 (14)	H10B—C10—H10C	109.00
C6—C7—C8	121.94 (14)	N2—C11—H11A	109.00
C7—C8—C9	120.39 (14)	N2—C11—H11B	109.00
N1—C9—C4	121.65 (13)	C2—C11—H11A	109.00
N1—C9—C8	119.58 (12)	C2—C11—H11B	109.00
C4—C9—C8	118.76 (13)	H11A—C11—H11B	108.00
N2—C11—C2	112.79 (12)	C13—C14—H14	120.00
O1—C12—N2	120.49 (14)	C15—C14—H14	120.00
O1—C12—C13	125.83 (14)	C14—C15—H15	120.00
N2—C12—C13	113.67 (12)	C16—C15—H15	120.00
C12—C13—C14	120.61 (14)	C15—C16—H16	120.00
C12—C13—C18	119.82 (13)	C17—C16—H16	120.00
C14—C13—C18	119.57 (14)	C16—C17—H17	120.00
C13—C14—C15	120.08 (16)	C18—C17—H17	120.00
C14—C15—C16	120.32 (17)	N2—C19—H19	117.00
C15—C16—C17	120.62 (19)	N3—C19—H19	117.00
			
C9—N1—C1—Cl1	−179.80 (10)	C3—C4—C9—N1	−0.4 (2)
C9—N1—C1—C2	0.3 (2)	C3—C4—C9—C8	178.79 (13)
C1—N1—C9—C4	0.4 (2)	C5—C4—C9—N1	−179.72 (13)
C1—N1—C9—C8	−178.82 (13)	C5—C4—C9—C8	−0.5 (2)
C12—N2—C11—C2	−69.62 (16)	C4—C5—C6—C7	0.0 (2)
C19—N2—C11—C2	114.68 (14)	C4—C5—C6—C10	179.76 (14)
C11—N2—C12—O1	−4.0 (2)	C5—C6—C7—C8	−0.5 (2)
C11—N2—C12—C13	174.94 (12)	C10—C6—C7—C8	179.74 (15)
C19—N2—C12—O1	171.64 (13)	C6—C7—C8—C9	0.5 (2)
C19—N2—C12—C13	−9.40 (18)	C7—C8—C9—N1	179.26 (14)
C11—N2—C19—N3	−179.97 (14)	C7—C8—C9—C4	0.1 (2)
C12—N2—C19—N3	4.5 (2)	O1—C12—C13—C14	7.1 (2)
C19—N3—C18—C13	−2.9 (2)	O1—C12—C13—C18	−172.61 (14)
C19—N3—C18—C17	176.95 (14)	N2—C12—C13—C14	−171.76 (13)
C18—N3—C19—N2	2.1 (2)	N2—C12—C13—C18	8.50 (19)
Cl1—C1—C2—C3	179.26 (11)	C12—C13—C14—C15	−179.62 (14)
Cl1—C1—C2—C11	−1.6 (2)	C18—C13—C14—C15	0.1 (2)
N1—C1—C2—C3	−0.8 (2)	C12—C13—C18—N3	−2.7 (2)
N1—C1—C2—C11	178.31 (14)	C12—C13—C18—C17	177.48 (13)
C1—C2—C3—C4	0.7 (2)	C14—C13—C18—N3	177.54 (13)
C11—C2—C3—C4	−178.43 (14)	C14—C13—C18—C17	−2.3 (2)
C1—C2—C11—N2	−75.60 (18)	C13—C14—C15—C16	1.7 (2)
C3—C2—C11—N2	103.49 (16)	C14—C15—C16—C17	−1.3 (3)
C2—C3—C4—C5	179.10 (14)	C15—C16—C17—C18	−0.9 (3)
C2—C3—C4—C9	−0.2 (2)	C16—C17—C18—N3	−177.16 (15)
C3—C4—C5—C6	−178.76 (14)	C16—C17—C18—C13	2.7 (2)
C9—C4—C5—C6	0.5 (2)		

Symmetry codes: (i) −*x*+1, −*y*+1, −*z*+2; (ii) *x*, −*y*+1/2, *z*+1/2; (iii) −*x*+1, −*y*, −*z*+2; (iv) −*x*+2, −*y*+1, −*z*+2; (v) *x*+1, −*y*+1/2, *z*+1/2; (vi) *x*, −*y*+1/2, *z*−1/2; (vii) −*x*+2, −*y*, −*z*+2; (viii) *x*, *y*−1, *z*; (ix) −*x*+1, *y*+1/2, −*z*+3/2; (x) *x*+1, *y*, *z*; (xi) −*x*+1, *y*−1/2, −*z*+3/2; (xii) *x*−1, *y*, *z*; (xiii) *x*−1, −*y*+1/2, *z*−1/2; (xiv) *x*, *y*+1, *z*.

### Hydrogen-bond geometry (Å, °)

**Table:**

Cg1 and Cg2 are the centroids of the N1/C1–C4/C9 and N2/N3/C12/C13/C18/C19 rings, respectively.

**Table:**

*D*—H···*A*	*D*—H	H···*A*	*D*···*A*	*D*—H···*A*
C19—H19···N3^iv^	0.93	2.51	3.271 (2)	139
C8—H8···Cg2^ii^	0.93	2.89	3.6598 (16)	142
C10—H10A···Cg1^iii^	0.96	2.68	3.5189 (17)	146

Symmetry codes: (iv) −*x*+2, −*y*+1, −*z*+2; (ii) *x*, −*y*+1/2, *z*+1/2; (iii) −*x*+1, −*y*, −*z*+2.
